# Antioxidants and Second Messengers of Free Radicals

**DOI:** 10.3390/antiox7110158

**Published:** 2018-11-06

**Authors:** Neven Zarkovic

**Affiliations:** Laboratory for Oxidative Stress (LabOS), Institute “Rudjer Boskovic”, HR-10000 Zagreb, Croatia; zarkovic@irb.hr

In the recent years, numerous research on the pathology of oxidative stress has been completed by intense studies on redox signaling implementing various experimental models and clinical trials. Nonetheless, there is still a lack of general understanding of pathophysiological roles played by reactive aldehydes like malondialdehyde, 4-hydroxynonenal, 4-hydroxyhexenal, acrolein, etc., which are considered as “second messengers of free radicals” [1,2]. Mostly being generated by lipid peroxidation, reactive aldehydes often form bioactive adducts with macromolecules that are important for the pathophysiology of living cells, thus, mimicking the effects of reactive oxygen species (ROS) even in the absence of severe oxidative stress [3,4,5]. Accordingly, we lack understanding on the complex effects of antioxidants that might be active in the regulation of toxic and/or hormetic effects of reactive aldehydes. This knowledge is necessary for better understanding of human physiology from the earliest days of life as well and for prevention and treatment of various stress- and age-associated diseases, which require integrative medicine treatment protocols [6,7].

This Special Issue collected original research papers and reviews on complex aspects of reactive aldehydes and their protein adducts generated during lipid peroxidation and their interference with natural and synthetic antioxidants in the physiology of cell and in the pathophysiology of various diseases, studied by modern bioanalytical methods applied in translational and integrative medicine.

Hence, focusing on the damaging effects of 4-hydroxynonenal (HNE) to the earliest events in our lives, i.e., the process of sperm-egg recognition, the Australian scientists reviewed the negative effects of HNE affecting the function and the stability of several germline proteins. Additionally, the authors pointed to the arachidonate 15-lipoxygenase (ALOX15) as a potential therapeutic target that could be exploited to protect human spermatozoa against oxidative stress [8]. They also prepared a very informative list of antioxidants tested for the improvement of male fertility summarizing their efficiencies.

On the other hand, focusing on the other end of the time-scale of human life, the Italian researchers wrote a very informative review on the relevance of reactive aldehydes in age-related disorders [9]. Reviewing the current knowledge on these complex topics of major relevance for modern biomedicine, the authors suggest that a major fraction of the toxic effects of oxidative stress observed in age-related disorders could depend on the formation of aldehyde-protein adducts (in particular, protein adducts of HNE and malondialdehyde (MDA). They also stressed the relevance of novel redox-proteomic approaches, which might reveal aldehydic modifications of distinct cellular proteins targeted in and after the course of oxidative stress, aiming to pave the way to targeted therapeutic strategies for age-associated disorders.

The importance of novel analytical approaches of redox-proteomics was also shown by researchers from Austria who described in their original research paper the method for detection of lipid-modified proteins that does not require an a priori knowledge on the chemical structure of lipid oxidation products or identification of their target proteins [10]. The method is based on the change of electrophoretic mobility of lipid-modified proteins, which is induced by conformational changes and cross-linking with other proteins. The authors have applied this method to successfully study the effects of oxidized palmitoyl-arachidonoyl-phosphatidylcholine (OxPAPC) on endothelial cells, identifying several known but also many new OxPAPC-binding proteins, thus presenting an important analytical breakthrough. This supports previous research by the Austrian pioneers in the field as Hermann Esterbauer and collaborators who discovered HNE, thus, constructing the fundaments for the modern scientific arena of lipid peroxidation [11]. 

The pathophysiological aspects of lipid peroxidation were further reviewed from two complementary aspects; by summarizing findings on HNE in redox homeostasis of gastrointestinal mucosa with possible implications for the stomach in health and in gastrointestinal diseases [12] and by reviewing options for modulation of oxidative stress and lipid peroxidation by endocannabinoids and their lipid analogues [13]. In the former article, the authors point to pathophysiological relevance of the HNE-protein adducts in digestive system of humans, especially stressing increased accumulation of HNE-modified proteins in gastric mucosa during infection and even after eradication of *H. pillory* infection. However, the authors of the later review paper suggest that a link between the endocannabinoid system (ECS) and redox homeostasis impairment could be crucial for cellular and tissue damages occurring in redox-dependent processes involving reactive oxygen and nitrogen species as well as lipid peroxidation-derived reactive aldehydes including acrolein, MDA and HNE. 

Consistent with that are the findings on the bioactivities or natural and synthetic antioxidants targeting reactive aldehydes as second messengers of free radicals in vitro or in vivo presented in the remaining papers of this Special Issue [14,15,16,17]. The authors of one review and two original papers were studying the structure-activity relations of particular plant extracts on their chemical composition. This might help us to better understand their activity principles [14,15,16], while in the last article of this Special Issue, the authors studied the relationship between antioxidant and growth regulating effects of synthetic chemical substances, notably of 1,4-dihydropyridine derivatives (DHS) [17]. Namely, various DHPs are known for their pleiotropic activity, some also act as antioxidants that are already used for UV-protection or as antihypertensive agents. In their original in vitro study using several well-known or newly synthesized DHPs to treat human osteoblast-like cells, the authors revealed some DHPs as possible therapeutic agents for osteoporosis. However, further research is needed to elucidate their bioactivity mechanisms in respect to signaling pathways involving HNE and related second messengers of free radicals [17].

Similarly, although working on a very different in vitro model of human skin cells treated with sea buckthorn seed oil, another group analyzed the effects of the particular oil on the redox balance and lipid metabolism in UV irradiated skin cells. This research aimed to examine whether the plant oil can have the UV-protective effect [16]. By doing so, the authors found beneficial effects of the buckthorn seed oil, which decreased the production of lipid peroxidation products (including HNE) simultaneously decreasing the cannabinoid receptor expression in UV-irradiated keratinocytes and fibroblasts.

Another in vitro study used several cell lines to test if HNE might be a relevant factor of beneficial effects of the widely used *Aloe vera* extracts (AV) [15]. This study found that the cell-type specific effects of AV, by itself was not toxic for any type of cells, while it modulated the cellular response to oxidative stress induced by hydrogen peroxide. Of particular relevance, it was found that high antioxidant levels of the AV did not interfere with enhanced cellular accumulation of the HNE-protein adducts in human endothelial cells, as revealed by the genuine cell-based ELISA specific for HNE-His, which was used for the first time. The authors concluded that these findings might help in understanding the activity principles of AV, particularly if used for the promotion of wound healing and/or for adjuvant cancer treatments.

Some options for the modulation of lipid peroxidation pathophysiology by plant extracts reach in antioxidants were eventually summarized in the review on the relationship between biological activities of such extracts and their chemical composition in the article focusing on the evening primrose extracts [14]. The authors of this review point to the biomedical use of the evening primrose oil (EPO) rich in linoleic acid (70–74%) and linolenic acid (8–10%), which are precursors of anti-inflammatory eicosanoids. Thus, EPO supplementation may result in an increase in plasma levels of linolenic acid and its metabolite dihomo-linolenic acid, which is oxidized by lipoxygenase (15-LOX) to 15-hydroxyeicosatrienoic acid (15-HETrE) or can be, under the influence of cyclooxygenase (COX), metabolized to series 1 prostaglandins, which exert anti-inflammatory and anti-proliferative properties. In addition, linolenic acid itself may suppress the production of inflammatory cytokines. Since linoleic acid is also a major source of HNE, one may assume that lipid peroxidation generating HNE could be also important for the multiple biological effects of EPO, as suggested for the *Aloe vera* extract in the paper described above [15].

In conclusion, more research is needed to evaluate, using advanced analytical methods and translation models, how the natural and/or synthetic antioxidants interfere with the pathophysiology of lipid peroxidation. Yet, by doing so, we could increase not only our understanding of this important field but also support the development of the modern integrative biomedicine for which both antioxidants and second messengers of free radicals, represented by HNE, are of highest importance. 
